# Fecal AAV8 shedding and co-housing reduces EAE disease severity: implications for preclinical study design

**DOI:** 10.3389/fmed.2026.1810215

**Published:** 2026-03-31

**Authors:** Cristina D. Gaddie, Geoffrey D. Keeler

**Affiliations:** Division of Cellular and Molecular Therapy, Department of Pediatrics, University of Florida College of Medicine, Gainesville, FL, United States

**Keywords:** AAV shedding, AAV viral genomes, experimental autoimmune encephalomyelitis, multiple sclerosis, pre-clinical variability

## Abstract

Adeno-Associated virus (AAV) vectors have become an invaluable tool in research and the clinic. Likewise, AAV is used in various animal models for investigating a host of diseases. However, AAV shedding, or the release of AAV particles from the body, remains an understudied field which could be detrimental to researchers and be a potential contributor to pre-clinical variability. To our knowledge, no studies have been performed to evaluate any relationship between AAV shed particles and the effects these particles may have on cage mates. As such, we evaluated the level of AAV viral genomes in the fecal matter of C57BL6/J mice following the intravenous administration of AAV8. Here we show that AAV genomes are shed from female C57BL/6J mice for a minimum of 2 months following intravenously injected AAV8 vector. We also sought to determine if these shed AAV particles could affect non-vectored cage mates in experimental autoimmune encephalomyelitis (EAE), an animal model for multiple sclerosis (MS). We found an association between caging vectored animals with non-vectored animals and a reduction in disease severity in the non-vectored animals. We also found an association between disease severity and the ratio of vectored:non-vectored animals in the cage. Our findings should act as a word of caution to researchers performing AAV studies and should be considered to prevent confounding variables and bias in results.

## Introduction

Adeno-Associated virus is one of the leading gene therapy platforms in use today. It has gained in popularity due, in part, to the wide range of tissues that can be targeted, while providing a favorable safety profile. AAV has been used in, or will be used, in over 300 clinical trials to date (Clinicaltrials.gov) and untold numbers of animal studies. AAV based therapies have shown clinical efficacy in 15 human diseases thus far. Further, the U.S. Food and Drug Administration (FDA) has approved seven AAV drugs to date with six currently on the market. Despite the clinical achievements, challenges remain. A lack of efficacy, or minimal efficacy, by AAV based therapies have been one of the driving forces for the use of high AAV doses in the clinic. Unfortunately, these high doses have led to severe adverse effects (SAEs) in many patients and culminated in 21 deaths thus far (1–16). In most, if not all cases, the lack of efficacy in trials has come as a shock given that these therapies are extremely effective in research animals.

Mouse models are the most common research animal used in pre-clinical studies when developing AAV gene therapies. Despite this, knowledge pertaining to AAV shedding in mouse models is lacking (17, 18). In the traditional sense, AAV shedding is the secretion of AAV virions through feces, urine, sweat, or any other bodily secretion following AAV administration. However, this definition is too restrictive and does not take into account the shedding of AAV genomes, which should be considered. It is well documented that mice exhibit coprophagy, the act of consuming feces. It is also common practice for AAV treated animals to be caged with non-treated animals. Despite this, there are currently no studies that have evaluated any effects associated with caging untreated animals with AAV treated animals.

In this work, we initially aimed to determine if AAV genomes are shed in the fecal matter of mice following intravenous administration of AAV8. For this we used digital droplet polymerase chain reaction (ddPCR) and determined that AAV transgene genomes are detectable in the fecal matter of treated animals for up to 2 months post AAV administration. Next, we sought to determine if caging AAV treated animals with non-treated animals altered disease course in untreated cage mates. To determine this, we utilized the animal model for Multiple Sclerosis, experimental autoimmune encephalomyelitis (EAE). We found that when AAV treated animals are caged with non-treated animals, there is a significant reduction in disease severity in the non-treated animals with the level of decreased severity being dependent on the ratio of treated:non-treated animals in a cage. Our findings represent a phenomenon that should be considered by researchers performing AAV studies in mice to prevent confounding results.

## Results

In previous work, we developed a liver-directed AAV gene therapy to treat multiple sclerosis in mice (19). We showed this AAV vector to be effective at preventing animals from developing EAE as well as reversing clinical signs of disease (19). We went on to show that this treatment was effective regardless of the encephalitogenic epitope used to induce disease or the genetic background of the animals used (20). For that work, cages always consisted of either AAV treated or non-treated animals due to *n*-values needed, caging requirements, and design of the study. However, given that fecal matter from MS patients could induce EAE in animals, and that mice are known to exhibit coprophagy, we postulated that AAV genomes could be shed through the stool and that consumption of this stool may modulate disease onset in EAE animals.

**FIGURE 1:**
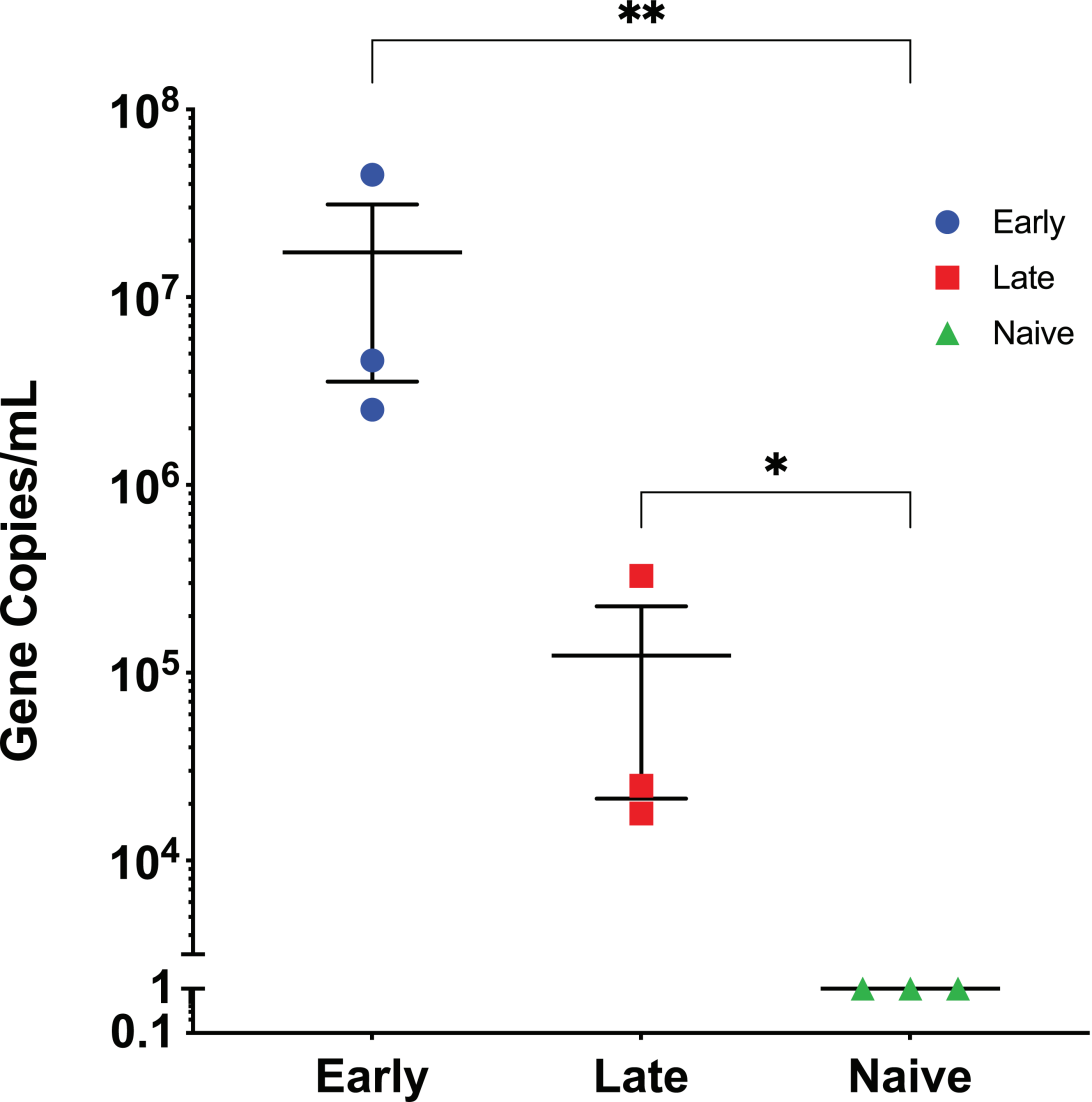
Quantification of AAV vector genomes in fecal matter. Animals were injected with 1 × 10^11^ vg of AAV8.MOG. Fecal matter was collected at early (10 days post vector administration) and late (2 months post vector administration) time points. Fecal matter was also collected from naïve animals as control. The level of MOG DNA was quantified via ddPCR. Fecal matter was found to contain significantly higher levels of MOG DNA at both the early and late timepoints as compared to naïve. Data are representative of 3 repeated experiments and are represented as mean ± SEM, *N* = 3, **p ≤ 0.005, *p ≤ 0.05, significance determined via Kruskal-Wallis test.

Initially, we determined the extent to which AAV vector genomes are shed through fecal matter of animals following i.v. injection of AAV. For this, female C57BL/6J mice were injected with 1 × 10^11^ vector genomes (vg) of an AAV8 vector encoding full-length myelin oligodendrocyte protein (MOG) under the control of a liver specific promoter (AAV8.MOG). For this work, MOG was chosen as a transgene based on previous work that showed liver-directed AAV MOG expression is effective at preventing and reversing EAE (19, 20). Fecal matter was collected directly from animals at 10 days and 2 months post AAV injection, DNA was extracted, and quantified via digital drop PCR (ddPCR) on a vg/mL basis using primers specific for MOG. To ensure consistency between animals, one fecal dropping was collected from each animal and dissolved in 1 mL of PBS. At 10 days post AAV administration, we found that an average of 1.73 × 10^7^ MOG vg/mL were detected (Figure 1). At 2 months following AAV administration, an average of 1.23 × 10^5^ vg/mL were detected (Figure 1). Both values were significantly higher than what was found in the fecal matter of naïve animals (*p* = 0.0036 and *p* = 0.0278, respectively) showing that AAV vector genomes are shed by animals for at least 2 months following intravenously administered AAV. This is in line with a recent report that showed intact viral genomes were detected in the fecal matter 33% of the time following intracranial injection of AAV8 while another study showed AAV genomes to be present in 100% of animals 2 days following AAV administration and persisting in 20% of animals for 14 days post administration (17, 18). Similarly, we find AAV genomes in the fecal 10 days post AAV administration. However, we also show AAV genomes to be present in the fecal matter up to 2 months post AAV administration. This difference is likely due to a differences in route of administration (one of the previous studies used intracranial injections), dose, AAV manufacturing techniques, technical differences in AAV administration, extraction efficiency, and our use of ddPCR for quantification, which is much more sensitive than the traditional qPCR used by previous studies. Thus, route of administration, dose, and techniques to evaluate results must be carefully considered when designing experiments.

**FIGURE 2:**
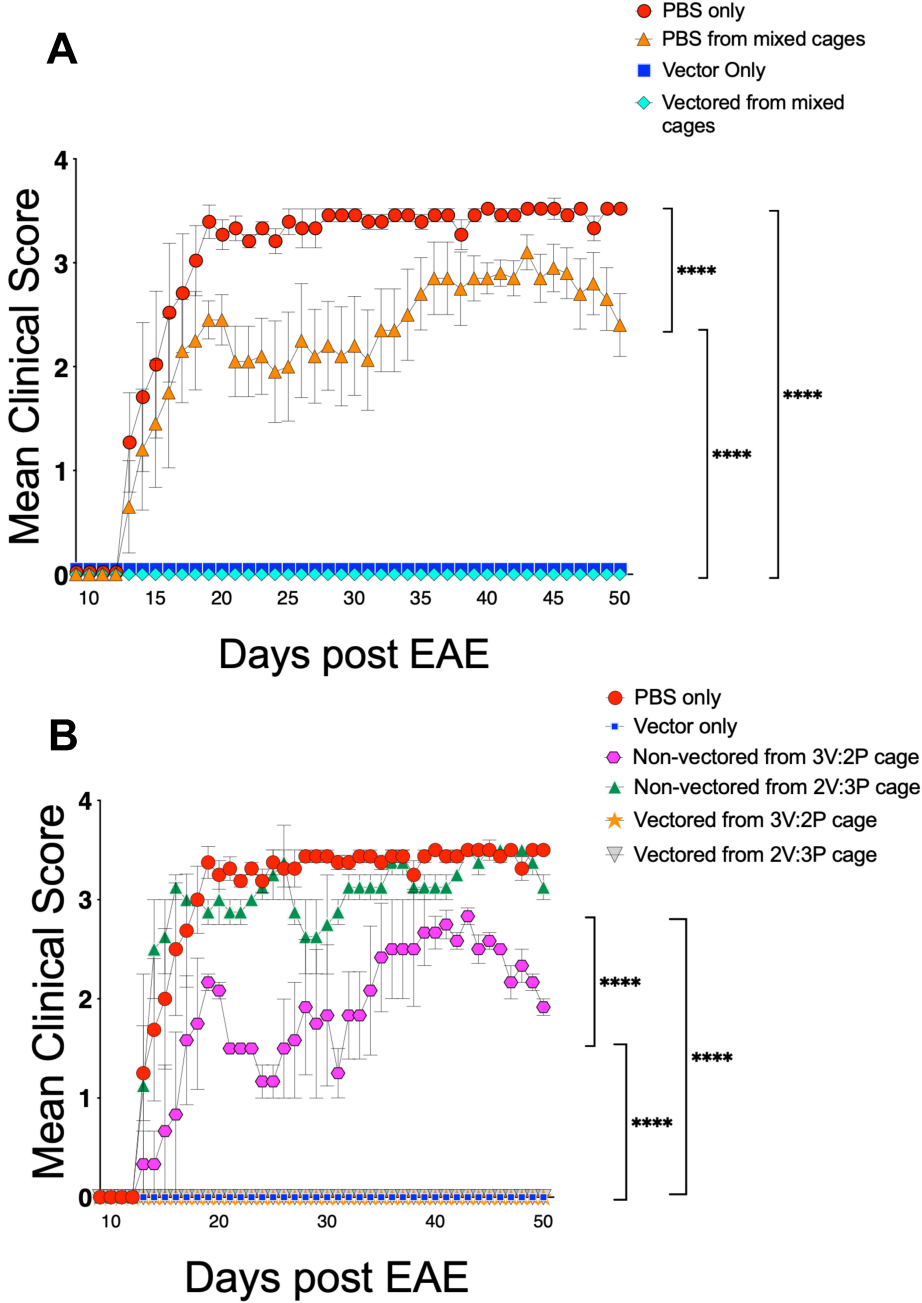
Effects of AAV shedding on disease course. Animals were injected with 1 × 10^11^ vg of AAV8.MOG. Two weeks later, Experimental Autoimmune Encephalomyelitis (EAE) was induced by injecting MOG_35–55_ emulsified in CFA, subcutaneously on the dorsal side of female mice. **(A)** Caging AAV treated and non-treated animals together results in reduced disease severity (Vector only and Vectored from mixed cages all remained at 0 for entirety of experiment), **(B)** correlation between the number of AAV vectored:non-vectored animals caged together and disease severity in non-vectored animals. (Vector only, Vectored from 3V:2P, and Vectored from 2V:3P all remained at 0 for entirety of experiment) (V = vectored animal, P = non-vectored animal) data are representative of 3 repeated experiments and are represented as mean ± SEM, *N* = 5 per group, ****p ≤ 0.0001, significance determined via Multiple Comparison Kruskal-Wallis test.

Next, we sought to determine if mixing AAV vectored animals with non-vectored animals would affect EAE severity. To determine this, animals were injected with 1 × 10^11^ vgs of AAV8.MOG vector into the tail veins of mice prior to disease induction. Two weeks following AAV vector administration, EAE was induced in female C57BL/6J mice by injecting MOG_35–55_ emulsified in CFA subcutaneously on the dorsal side. Animals were then monitored daily and mean clinical score (MCS) determined based on a 5 point scale (Table 1). To determine if the vector genomes that are shed in the fecal matter of AAV treated animals had any effect on disease progression and severity, animals were caged in 4 different groups: (1) All animals in the cage received AAV (Vector only), (2) no animals in the cage received AAV (PBS only), (3) 2 animals received AAV and 3 animals did not (3P:2V), (4) 3 animals were treated with AAV and 2 animals were untreated (2P:3V). What we found was that non-vectored animals that were housed with vectored cage mates, when pooled together, developed a less severe disease course as compared to control EAE animals that were not housed with vectored cage mates (Figure 2A). Interestingly, when compared separately, we found that the PBS animals from the cage where 2 animals were vectored and 3 were non-vectored showed no significant difference in disease severity as compared to EAE only controls (Figure 2B). However, when 3 animals in the cage receive AAV, the non-vectored animals present with a delayed disease onset and reduced disease severity as compared to EAE only controls (Figure 2B). This suggests there may to be a threshold that needs to be met for protection to be conferred, though this needs further investigation to be confirmed.

**TABLE 1:** Experimental autoimmune encephalomyelitis (EAE) mean clinical scores.

Mean clinical score	Disease symptom
0	No signs of disease/appear naive
0.5	Tip of tail paralyzed/limp
1	Entire tail paralyzed/limp
1.5	Tail paralysis with reduced grip
2.0	Knuckling exhibited in one hind foot
2.5	Knuckling exhibited in both hind feet
3.0	Paralysis in both hind limbs
3.5	Hind limb paralysis and unable to right itself
4.0	Complete hind-limb paralysis and partial front-limb paralysis
5.0	Moribund

## Discussion

In this work we performed ddPCR and showed that AAV vector genomes are passed in the fecal matter of mice. Further, we show that non-vectored animals caged with vectored animals exhibit reduced EAE disease severity, presumably due to the consumption of fecal matter from treated animals. While the mechanisms of this effect are unclear, we have two possible hypotheses as to how this may be occurring. First, the AAV genomes being consumed by non-treated animals may be inducing oral tolerance to the AAV transgene. Traditionally, oral tolerance is defined as induction of immune tolerance, where responses to a specific antigen are systemically suppressed, following the uptake of an orally administered protein by the intestinal immune system (21). More recently it has been shown that ingestion of nucleic acids can promote oral tolerance induction through expansion of intraepithelial lymphocytes (22). Further, when DNA is ingested, a much lower antigenic threshold is needed for oral tolerance to be established. Thus, we speculate that one possible mechanism for what is seen in this study is that along with the DNA, small amounts of transgene (in this case MOG) are also being ingested and inducing oral tolerance.

Secondly, microbiome transfer cannot be ruled out as a possible explanation for the results seen here. Of interest here, the establishment of the microbiota-gut-brain axis has illuminated a pathway of communication between the gut microbiome and the central nervous system (CNS). Though the mechanisms through which the gut and CNS communicate remain unclear, there is evidence building that the gut may play a major role in the progression of Multiple Sclerosis (MS) (19–23). Much of the evidence for an imperative role of the gut microbiome in regulating MS has come from the EAE animal model used to study MS. In a T cell receptor (TCR) transgenic mouse carrying a TCR specific for myelin oligodendrocyte glycoprotein (MOG) peptide, nearly all mice raised in specific pathogen-free (SPF) conditions develop spontaneous relapsing-remitting EAE while those raised in a germ-free environment, do not develop EAE (24). Interestingly, mice raised in a germ-free environment developed disease following exposure to fecal matter from SPF raised mice (25). Similarly, it has been shown that transferring the gut microbiota from a patient suffering from MS to a mouse, results in the spontaneous development of EAE (19). Another study identified specific bacteria in the gut of patients suffering from MS that are responsible for modulating T cell responses and contribute to inflammation (26). Finally, the commensal gut flora, in the absence of pathogenic agents, may be responsible for inducing the immune responses that lead to the onset of relapsing-remitting EAE (25). Alternatively, there may be an unexplored relationship between AAV administration and the gut microbiome. Further research is needed to identify the mechanism that is responsible for the results seen in this study.

Despite the large number of AAV studies being conducted and the integral role of the microbiome in life, there are no studies we are aware of that have investigated any relationship between AAV administration and the microbiome. Further, as far as we know, no one has investigated any link that may exist between shed AAV particles and the effects they may have on the disease state of cage mates. In this work, we provide evidence that shed AAV particles may result in reduced disease severity in the EAE model.

We would be remiss if we did not acknowledge that this is a small study with limitations and areas that need further investigation. Here we only investigated one AAV serotype and one transgene. Further, we only investigated one animal model. It is not clear if these results are specific to this model/transgene combination or if they will transcend other AAV experiments. It also remains unclear if active virus, protein, or only AAV vector genomes are passed in the fecal matter and if these are present in cage mates. There is also a need for large scale studies and germ free environments as well as immune phenotyping to pinpoint the exact mechanisms for what is seen here. Despite the limitations of the current study, we feel it is prudent that the results be considered by all researchers using AAV to prevent confounding variables and bias in results, thus potentially reducing pre-clinical variability.

## Materials and methods

### Mice, vector administration, and EAE induction

For this work, 9–10 weeks old C57BL/6J mice (The Jackson Laboratory, Bar Harbor, ME) were used. All mice were provided with food and water, *ad libitum*, were subjected to a 14 h light/10 h dark cycle and were housed in a pathogen free environment. Animals were injected with 1 × 10^11^ vg via the tail vein 2 weeks prior to EAE induction. Complete Freund’s Adjuvant was made by adding 10 mg/mL *Mycobacterium tuberculosis* (Difco Laboratioes, Franklin Lakes, NJ). For EAE induction, an emulsion was made by mixing MOG_35–55_ peptide (Genscript, Piscataway, NJ) with Complete Freund’s Adjuvant (Sigma Aldrich, St Louis, MO). The emulsion was injected subcutaneously over two injection sites on the dorsal side. Two and twenty-four hours following emulsion injection, pertussis toxin (Hooke Laboratories, Lawrence, MA) was injected intraperitoneally. Animals were checked and MCS scored daily starting at 7 days post EAE induction. Animals were randomly chosen for treatment group on a rolling enrollment basis and all animals were scored in a blinded fashion. All studies involving animals were performed in accordance with the guidelines of the University of Florida Institutional Animal Care and Use Committee (IACUC).

### Vector production

A recombinant AAV8 vector containing the full length MOG sequence under the control of a ApoE.hAAT promoter was produced in human embryonic kidney (HEK293) cells using a three plasmid system as previously described (27). After 72 h, cells and media were harvested and virus purified by iodixanol gradient and titers were determined via digital drop PCR as previously described (28, 29).

### Fecal matter collection and quantification

Fecal matter was collected from individual animals in sterile, pyrogen free, nuclease free, micro centrifuge tubes. Fecal matter was dissolved in 1 mL of PBS. DNA was extracted using a DNeasy extraction kit (Qiagen, Hilden, Germany) and standard protocol. DNA was eluted in 200 uL and quantified via ddPCR by the ICBR Gene Expression & Genotyping Core at the University of Florida, as previously described (29). Briefly, purified vectors were diluted 50-fold with DNase I to remove exogenous DNA. DNase I was then deactivated, viral capsids broken via proteinase K. Viral DNA was purified and resuspended in TE and diluted to 0.005 ng/μL. For ddPCR, a gradient test and melting curve were performed to optimize annealing/extension temperatures and target specific amplification. Non-template controls were also run to determine cleanliness of reagents and workflow. All samples were run in duplicate and average copies/μL were determined.

### Statistical analysis

All results were analyzed using GraphPad Prism software (La Jolla, CA). Significance was determined via a multiple comparison Kruskal-Wallis test, and data are presented as mean ± SEM with ****p ≤ 0.0001, **p ≤ 0.005, *p ≤ 0.05, for EAE experiments.

## Data Availability

The original contributions presented in this study are included in this article/supplementary material, further inquiries can be directed to the corresponding author.
